# Antimicrobial Peptides of Salmonid Fish: From Form to Function

**DOI:** 10.3390/biology9080233

**Published:** 2020-08-18

**Authors:** Sascha R. Brunner, Joseph F. A. Varga, Brian Dixon

**Affiliations:** 1 Department of Biology, University of Waterloo, Waterloo, ON N2L 3G1, Canada; s2brunner@uwaterloo.ca (S.R.B.); joseph.varga@uwaterloo.ca (J.F.A.V.); 2 Groningen Institute for Evolutionary Life Sciences, University of Groningen, 9747 AG Groningen, The Netherlands

**Keywords:** teleost, aquaculture, rainbow trout, host defense, fish immunology, immunomodulation, AMP genes

## Abstract

Antimicrobial peptides (AMPs) are small, usually cationic, and amphiphilic molecules that play a crucial role in molecular and cellular host defense against pathogens, tissue damage, and infection. AMPs are present in all metazoans and several have been discovered in teleosts. Some teleosts, such as salmonids, have undergone whole genome duplication events and retained a diverse AMP repertoire. Salmonid AMPs have also been shown to possess diverse and potent antibacterial, antiviral, and antiparasitic activity and are induced by a variety of factors, including dietary components and specific molecules also known as pathogen-associated molecular patterns (PAMPs), which may activate downstream signals to initiate transcription of AMP genes. Moreover, a multitude of cell lines have been established from various salmonid species, making it possible to study host-pathogen interactions in vitro, and several of these cell lines have been shown to express various AMPs. In this review, the structure, function, transcriptional regulation, and immunomodulatory role of salmonid AMPs are highlighted in health and disease. It is important to characterize and understand how salmonid AMPs function as this may lead to a better understanding of host-pathogen interactions with implications for aquaculture and medicine.

## 1. Introduction

Salmonids belong to the Salmonidae family of fish and serve as excellent models for experiments in modern biology [1,2]. Salmonids have undergone a whole genome duplication, approximately 70–100 million years ago [1]. Throughout this time, they have developed an intricate immune system, equipped to tackle harmful pathogens, that is still poorly understood. Attempting to better characterize the immune systems of salmonids is of great interest, as they are one of the most successful aquaculture species with growing demand and production [3,4]. The immune system of fish, like mammals, can be divided into innate and adaptive arms, where the innate branch serves a role in recognizing non-self and danger signals and is generally more acute, while the adaptive branch is concerned with long-term immunity [5]. The innate immune system can be further broken down into physical, cellular, and humoral components [5]. However, in most cases, the innate and adaptive systems work more as a cohesive unit, to neutralize a potential threat. Antimicrobial peptides (AMPs) which are small, usually cationic, and amphiphilic molecules that play a role in molecular host defense by interacting with negatively charged components of pathogens or binding to cell surface receptors on host cells [6,7,8]. AMPs are crucial components of teleost innate and adaptive immunity [6,7,8]. Besides playing an essential role in immunity, many fish AMPs are being considered as potential alternative therapeutics against pathogens in which antibiotic resistance is on the rise [9]. In general, fish AMPs may be categorized into five different classes based on their structure: β-defensins, cathelicidins, hepcidins, histone-derived peptides, and piscidins [7,8]. However, AMPs may also arise from fragments of any protein, which may not necessarily fall into these classes. The evolutionary relationship among salmonid cathelicidins and hepcidins was constructed as part of this review (Figures S1 and S2). Salmonid AMPs are diverse molecules with a relatively well-studied role in salmonid health and disease [7,8]. However, it is likely that some salmonid AMPs with uncharacterized structure and function remain to be discovered, which may lead to novel therapeutics against fish pathogens. Continuing to investigate AMPs in salmonid species will help facilitate these important discoveries.

## 2. Structural Properties of Salmonid AMPs

### 2.1. Length

The length of AMPs is an important structural property that may affect function. Extending and fragmenting the length of select AMPs has led to increased antimicrobial activity [6]. However, this has been understudied in salmonid-derived AMPs. In general, the length of AMPs is 12–50 amino acids [10,11]. However, salmonid AMPs range from 11 to 79 amino acids (Table S1). The average length of salmonid AMPs in The Antimicrobial Peptide Database (APD) and Data Repository of Antimicrobial Peptides (DRAMP) is 41 amino acids (Table S1). Most salmonid AMPs in these databases were discovered in rainbow trout (*Oncorhynchus mykiss*). Evaluating the antimicrobial properties of fragments from longer AMPs, such as Oncorhyncin II, and from different species may provide insight into the length effect observed in mammalian AMPs. Fragments of other teleost AMPs have also been studied in this context and were shown to retain antimicrobial activity; therefore, it is likely that this phenomenon is conserved in salmonids as well and will be reviewed here [6,12].

### 2.2. Charge

A key factor that directly influences the antimicrobial activity of AMPs is charge. In general, AMPs are positively charged molecules [13]. The effects of charge on salmonid AMPs have not been investigated. However, the net charge of salmonid AMPs is diverse and ranges from 0 to 30 [12]. Interestingly, Histone H2A, an AMP from rainbow trout with a neutral charge of zero, possesses potent antibacterial activity against a wide array of bacteria [13,14]. Suggesting that, although charge is an important factor in directing AMP activity, it is not essential. The salmonid AMP with the highest net charge (+30) is Oncorhyncin II, also from rainbow trout [15]. Although the peptides share antimicrobial efficacy among the select bacterial species, H2A appears to be slightly more effective than Oncorhyncin II against *Planococcus citreus*, but less effective than Oncorhynchin II against *Micrococcus luteus* [16]. To date, Histone H2A is the only known non-cationic AMP from salmonids and only a handful have been discovered among fish [17]. Chemokine 11 (Ck11) is another potent AMP from rainbow trout and was the first fish chemokine discovered to possess antimicrobial activity [17]. Chemokines are a large family of proteins, each with four invariant cysteines, and can be subdivided into major and minor subfamilies based on the sequence surrounding the first two cysteines [16]. Besides aiding in classification, the cysteines also impart a positive charge on chemokines, which is important to their functional roles in binding negatively charged receptors and directing the migration of immune and nonimmune cells to specialized areas within organisms [18,19,20]. A predictive model of Ck11 revealed similarities to some human chemokines, namely C-C motif chemokine ligand 27 (CCL27) and C-C motif chemokine ligand 28 (CCL28), but Ck11 appears to have more uncharged patches on its surface than CCL28 [21]. Even though several salmonid chemokines have been identified, their antimicrobial properties remain unknown [22,23]. A lower charge may contribute to reduced AMP activity, as observed among human chemokines, but this remains to be investigated in salmonids [22].

### 2.3. Hydrophobicity

Hydrophobicity is another important characteristic of AMPs and can have significant effects on peptide function [22,23]. A hydrophobicity window has been established for some AMPs, and increasing or decreasing hydrophobicity beyond this range results in dramatically impaired antimicrobial activity [24]. Increasing the hydrophobicity of peptides has been observed to increase hemolytic activity [24]. Although the direct effects of hydrophobicity on salmonid AMPs are unknown, hemolysis in the context of salmonid AMPs has been studied to some extent [12]. The hemolytic effect of Atlantic salmon (*Salmo salar*) cathelicidin 1 (Cath1) and 2 (Cath2) was tested on Atlantic salmon erythrocytes. Although Cath2 did not show any hemolytic activity, Cath1 was extremely hemolytic [24]. The APD defined total hydrophobic ratio for each AMP sequence is 8% and 22%, respectively [12,25]. However, when tested in the presence of serum from Atlantic salmon, the hemolytic activity of Cath1 was abolished [13]. A fragment of rainbow trout cathelicidin 1 (Cath1) was also evaluated for hemolytic activity against rainbow trout erythrocytes and, although this fragment has a total hydrophobic ratio of 27%, it had a slight hemolytic activity compared with the control [12]. Another rainbow trout-derived AMP, histone H2A, shows some hemolytic activity against rainbow trout erythrocytes beyond 0.3 µM [26]. Histone H2A is quite hydrophobic in nature, with a total hydrophobic ratio of 50% [27]. Albeit, even with a hydrophobicity ratio of 50%, it does not lead to complete hemolysis. Collectively, these findings suggest that AMP hydrophobicity may contribute to hemolysis in fish erythrocytes, as documented in the mammalian literature, but further studies are needed.

## 3. Function of Salmonid AMPs

### 3.1. Antibacterial

Some of the most important fish pathogens are bacteria. Thus, studying direct antibacterial activity of AMPs in vitro and in vivo is crucial to the understanding of how these AMPs act in the innate immune response. Truncated rainbow trout cathelicidin 1 (Cath1) was found to inhibit the growth of six Gram-positive and Gram-negative bacteria species including *Escherichia coli* (*E. coli*), *Aeromonas salmonicida* (*A. salmonicida*), *Photobacterium damselae* (*P. damselae*), *Vibrio anguillarum* (*V. anguillarum*), *Yersinia ruckeri* (*Y. ruckeri*), and *Lactococcus garvieae* (*L. garvieae*); the minimal inhibitory concentration (MIC) against these bacteria was found to range from 0.1 µM to 10 µM [24]. Interestingly, truncated Atlantic salmon cathelicidins 1 and 2 were found to be antibacterial against *V. anguillarum* at relatively low MICs (5 and 10 µM, respectively); however, more than 80 µM of each peptide [24] was required to inhibit *Y. ruckeri* [24]. This is surprising as both cathelicidins were found to be significantly upregulated during yersiniosis [27]. Moreover, cleavage of the cathelicidins is vital to the direct killing ability on some pathogens as full-length Cath2 did not show significant antibacterial activity against *V. anguillarum* (up to 40 µM), but did so against human pathogens *Salmonella typhimurium* (*S. typhimurium*) or *Staphyloccocus aureus* (*S. aureus*) [27].

D’Este et al. [27] tested the antibacterial activity of predicted and alternative C-terminal active cathelicidin AMPs of brown trout (*Salmo trutta*), rainbow trout, and grayling (*Thymallus thymallus*) against a panel of bacteria [27]. Notably, the longer grayling Cath2 synthetic fragment showed potent activity against *E. coli*, *A. salmonicida*, and Gram-positive *L. garvieae*, while a shorter fragment did to a lesser extent, indicating that peptide length is an important factor for antibacterial function [26,27]. Similarly, N-terminus truncated rainbow trout Cath2 showed less direct AMP activity than the non-truncated version [28]. Moreover, salt concentration of the medium significantly affected the function of salmonid cathelicidins, with higher salt concentrations coinciding with lower membrane permeabilization activity [29]. This salt concentration dependency is not found in other salmonid AMP families [29]. These results raise questions about whether salmonid cathelicidins do indeed possess AMP activity in physiological conditions in vivo, unless other beneficial components are present, which are difficult to replicate in vitro.

Brown trout hepcidin (Hepc) together with a scrambled version of this peptide was tested for its antibacterial activities in a disc diffusion assay [30]. Both peptides were able to inhibit the growth of four Gram-negative *Acinetobacter baumannii* (*A. baumannii*), *A. salmonicida*, *Aeromonas hydrophila* (*A. hydrophila*), and *Pseudomonas aeruginosa* (*P. aeruginosa*), as well as two Gram-positive bacteria *S. aureus* and *Enterococcus faecium* (*E. faecium*), at a concentration of 2 mg/mL [30]. The scrambled peptide showed significantly less inhibition activity against all bacteria tested compared with the synthetic mature peptide, indicating that the sequence order is an important factor determining the antibacterial potency of this peptide [30]. In a similar assay, Hepc from Siberian taimen (*Hucho taimen*) was able to inhibit the growth of Gram-negative *E. coli* and *S. aureus*, and Gram-positive *Micrococcus lsyodeikticus* (*M. lysodeikticus*) [31]. In addition, rainbow trout oxidized Hepc has been found to possess direct antibacterial activity against *E. coli* and the Gram-negative *Piscirickettsia salmonis* (*P. salmonis*), the causative agent of piscirickettsiosis, at an MIC of 33.5 µM [32]. Moreover, Z-stack confocal microscopy in combination with a Sytox membrane permeabilization assay revealed a translocation of oxidized rainbow trout Hepc into *P. salmonis* cells without disrupting the bacterial membrane, indicating that direct membrane disruption is likely not the mechanism of action for this AMP [32]. In contrast, another study including brown trout Hepc suggests that this peptide does indeed act through a membrane permeabilization mechanism [30]. To more clearly elucidate the role of Hepc in the immune response of a salmonid species, the effect of *P. salmonis* infection on hepcidin (*hepc*) expression in the RTS-11 cell line was compared against a control containing heat-inactivated *P. salmonis* [32]. RTS11 cells at 12 h post-infection (hpi) with *P. salmonis* were stained with specific anti-*P. salmonis* or anti-Hepc antisera to visualize co-localization with phagosome containing bacteria. Co-localization of vesicles containing Hepc and heat-inactivated *P. salmonis* control cells did occur. However, no co-localization was observed with live *P. salmonis*. The vesicles containing the antimicrobial peptide were unable to merge with the infective bacteria, indicating *P. salmonis* is able to inhibit phagosome-lysosome fusion [32].

Histone AMP H2A that was extracted from skin exudates of rainbow trout displayed strong antimicrobial activity against Gram-positive bacteria in the submolecular range [13]. Further kinetic analysis revealed that inhibition of bacteria occurred as early as 30 minutes post-incubation [13]. Similar ranges were found against several Gram-negative and Gram-positive bacteria using Oncorhyncin III, an AMP derived from the non-histone chromosomal protein H6 [13].

Lastly, salmine, an AMP derived from salmon milt tissues, has been shown to inhibit *Listeria monocytogenes* (*L. monocytogenes*), a Gram-positive bacterium causing listeriosis in humans when contaminated smoked salmon is consumed, at MICs of 0.5–1 mg/mL [33,34]. Cheng et al. [34] evaluated its applicability for the food industry and did not find an inhibition; however, a delay of the log phase by ~6 days was observed when *L. monocytogenes* grown on smoked salmon pieces were treated with 5 mg/g of salmine [34]. This suggests that salmine might be bacteriostatic, at least temporarily, against *L. monocytogenes*.

### 3.2. Antiviral

Evidence of direct antiviral activity of salmonid AMPs is rare. Epithelioma papulosum cyprini (EPC) cells transfected with a plasmid containing a recombinant rainbow trout β-defensin 1 (Defb1) have gained immunity to the viral haemorrhagic septicaemia virus (VHSV) [35]. The infectivity of VHSV was reduced 80–90%, compared with control cells transfected with the plasmid alone [35]. This immunity could be conferred to other EPC cells by transfer of the surrounding medium, indicating a possible upregulation of antiviral agents such as interferon type 1 (IFN type 1) [7]. However, further research needs to be conducted to test this hypothesis. Moreover, whether this peptide is able to upregulate the IFN type 1 response in salmonids in vitro and in vivo needs to be further investigated.

### 3.3. Anti-Oomycete and Antifungal

While the antibacterial activities of salmonid AMPs have been studied extensively, their direct antifungal abilities were mostly overlooked. Synthetic rainbow trout Cath2, aside from direct antibacterial abilities, exhibits antifungal properties as well in the form of a delay of sporulation of *Saprolegnia parasitica* (*S. parasitica*) cysts at concentrations of 50 µM and 100 µM [36]. This effect was deemed to be specific to Cath2 and not owing to the high concentration of peptide alone, as two other synthetic peptides derived from trout Il-17a and Il-22 were not found to delay sporulation of *S. parasitica* at a concentration of 100 µM [36]. Synthetic brown trout Hepc and a scrambled version of the same peptide were tested against the haploid yeast *Candida glabrata* (*C. glabrata*) in a disc diffusion assay [30]. Both peptides were able to inhibit the growth of *C. glabrata*, however, the inhibition area around disc containing the predicted Hepc was significantly larger than around the scrambled peptide at a concentration of 2 mg/mL [30]. Finally, histone H2A isolated from rainbow trout skin exudates showed potent antifungal activity against the yeast *Saccharomyces cerevisiae* (*S. cerevisiae*) at MICs as low as 0.3 µM [13].

## 4. Factors Affecting Salmonid AMP Gene Expression

### 4.1. Bacterial Pathogens

The AMP response to bacterial pathogens in salmonids and other teleosts has been well studied. For the purpose of this review, the interactions between pathogen and AMP gene expression will be discussed, while focusing on well characterized AMPs, namely, the hepcidin, cathelicidin, and defensin families. In Atlantic salmon (*Salmo salar*) liver tissue, *hepc* significantly increased 24 h post-bacterial challenge with a genetically attenuated strain of *A. salmonicida* [37]. However, in fish starved prior to the bacterial challenge for 28 days, the increase in *hepc* gene expression was more profound [37]. Starvation also led to several changes in the immune system including reduced immune and plasma gene expression [37]. The reason for this remains unknown, but *A. salmonicida* and *A. hydrophila* also increase the expression of *hepc* in various immune tissues of sharp-snouted lenok (*Brachymystax lenok*) and brown trout [13,25]. A plethora of AMPs, including *hepc*, are differentially expressed in immune organs such as the spleen and head kidney of rainbow trout infected with *L. garvieae* [38]. Interestingly, in silico target prediction of the spleen and head kidney of Atlantic salmon during infection with *P. salmonis*, an intracellular bacterium, revealed *hepc1* as a target of select miRNAs [39]. Furthermore, the expression of *hepc1* was predicted to increase as mir-143_29 decreased during *P. salmonis* infection [39]. This suggests that miRNAs induced by bacterial pathogens may fine-tune the AMP response during infections. However, this is yet to be investigated in salmonids physiologically.

Cathelicidins are also very well studied across salmonids and their expression has been detected in diverse tissues and organs. For example, cathelicidin has been detected in the skin, gills, liver, pyloric caeca, intestine, spleen, kidney, brain, heart, and muscle of Arctic char (*Salvelinus alpinus*) and the gene expression of cathelicidin (*cath*) appears to be highest in the skin, pyloric caeca, spleen, kidney, and heart of fish [40]. However, when Arctic char are infected with *A. salmonicida*, the expression of *cath* mRNA appears to increase in all aforementioned tissues [40]. In other salmonids, such as rainbow trout, injection with formalin-inactivated A. salmonicidia rapidly increases the expression of *cath1* and *cath2* mRNA in the head kidney, spleen, intestine, and muscle [41]. Formalin-inactivation of *Y. ruckeri*, *L. graviae*, and *F. psychrophilum* also induces rapid expression of *cath1* and *cath2* mRNA in these tissues [41]. This suggests that, in rainbow trout, AMP gene expression patterns within the first 24 h after exposure to various bacterial pathogens are conserved among tissues [41]. This also signifies that AMP gene expression is stimulated via similar upstream signaling pathways that are yet to be elucidated in salmonids. However, variations exist in the intensity and late AMP gene expression responses to bacterial pathogen inoculation [42]. Under normal circumstances, the basal level of *cath* mRNA expression among the head kidney, spleen, intestine, and muscle tissue from healthy rainbow trout remains relatively low [41]. Cathelicidin 2 has also been detected in the blood of healthy fish and fish infected with bacteria, suggesting that blood cell populations also express cathelicidins as they circulate through the blood [42]. In addition to this, head kidney macrophages from rainbow trout have also been shown to express cathelicidins [42,43]. As bacteria may increase AMP gene expression, exposure to inactivated or live bacteria may be necessary to study the AMP response. To date, few studies have examined transcriptional regulation of AMPs in salmonids, but exposure of primary head kidney macrophages from rainbow trout to recombinant tumor necrosis factor-α (Tnfa3) has been shown to increase AMPs, including cathelicidin 1 and cathelicidin 2 [44]. Moreover, Tnfa3 is induced via lipopolysaccharide (LPS), a major component of the outer membrane of Gram-negative bacteria such as *Y. ruckeri* [44]. RTL cells stimulated with rainbow trout recombinant interleukin 1 beta 1 (Il1b1) protein for 4 h exhibit a significant increase in both *il1b1* and *cath2* mRNA [36]. *Cath1* was also detected in the Ilb1 treated cells, but the level of expression could not be quantified [36]. These data suggest that pro-inflammatory cytokines may stimulate cathelicidin gene expression in salmonids. It is important to dissect the molecular mechanisms surrounding AMP gene expression in salmonids to better understand AMP-induced immune responses to bacterial pathogens.

Defensins are also a well studied family of salmonid AMPs and most of them have been discovered within the last few decades [35,43,45]. However, studies are lacking regarding defensin gene expression in salmonids during bacterial infection and are mostly limited to *Y. ruckeri*, the causative agent of enteric redmouth disease [46]. *Y. ruckeri* infection of rainbow trout increases the expression of *defb2* mRNA within the gut tissue, while *defb3* mRNA increases in the gill [43]. *Y. ruckeri* infection via injection or cohabitation, in rainbow trout, also results in a marked upregulation of *defb3* and *defb4* mRNA in the blood of infected animals compared with controls [42]. Interestingly, interleukin 22 (*il22*) mRNA transcripts were also found to be upregulated in the same animals [42]. The expression of *il22* in normal healthy rainbow trout spleen tissue is relatively low compared with other tissues [47]. However, when rainbow trout splenocytes are stimulated with recombinant Il-22 protein, the expression of *defb3* and *defb4* mRNA rapidly increases [47]. This suggests that Il-22 may play an important role in regulating the expression of defensin genes in salmonids. However, exposure of rainbow trout B cells to LPS or heat-killed *A. salmonicida* did not result in the upregulation of *defb3* [48]. On the other hand, phagocytosis increases *defb3* mRNA in specific subsets of phagocytic B cells, such as IgT+ B cells from the head kidney [48]. Therefore, the defensin AMP gene response may be specific to either the bacterial pathogen, cell type, or even the state of the cell, responding to the threat. It is not surprising that most of the research thus far has focused on major bacterial pathogens of aquaculture significance, but whether other bacterial pathogens or commensal microbes influence AMP gene expression in salmonids remains unknown.

### 4.2. Viral Pathogens

Salmonids are susceptible to a wide range of viruses that cause high mortality, and the antiviral responses are relatively well understood; these topics have been excellently reviewed elsewhere [49,50]. Infection of brown trout with viral hemorrhagic septicemia virus (VHSV) led to an increase in *defb3*, *cath1*, and *hepc1* gene expression in the kidney [51]. Interestingly, *defb3* was upregulated before the other AMPs and it was also elevated at days 7 and 16 post-infection [51]. Animals claimed to be resistant by the authors, which did not test positive for VHSV, also had elevated levels of *defb3* in the kidney [51], suggesting that the AMP may confer protection against VHSV. VHSV has also been shown to increase the expression of *hepc* in rainbow trout liver; however, the gene expression of other AMPs such as *ck11* and liver expressed antimicrobial peptide 2 (*leap2a*) did not change significantly at 5 days post-infection (dpi) [52].

Red blood cells have also been shown to participate in salmonid immune responses during viral infection [53]. For example, red blood cells from rainbow trout exposed to VHSV showed an upregulation of Defb1 at the protein level, while Hepc protein levels remained unchanged compared with time-matched controls [53]. The fact that protein levels increase suggests that some AMPs in salmonids may be able to evade VHSV-mediated host translational repression [54,55]. Using pooled head kidney and spleen tissue from VHSV infected rainbow trout fingerlings, Chinchilla et al. demonstrated that the virus can downregulate the expression of *ck11*, but upregulate the expression of *hepc* [56]. Besides modulating the expression of AMPs, VHSV infection also decreased the expression of several interleukins, a subgroup of cytokines, that play a crucial role in mediating teleost immune responses [57,58]. A microarray analysis of RTS-11 cells treated with recombinant interleukin-1beta (Il-1b), a potent proinflammatory cytokine, revealed that Il-1b also leads to a rapid increase *hepc* gene expression [59]. Therefore, red blood cells and some leukocytes may play a key role in regulating defense genes, such as AMPs, during viral infection in rainbow trout.

Another important viral pathogen that infects salmonids is infectious hematopoietic necrosis virus (IHNV); this virus decreases the expression of *def3* in rainbow trout spleen, but not significantly [60]. However, the role of *defb3* in IHNV infection remains unknown. The activities of defensins on viral infection in mammalian studies have been well documented, but their role remains controversial, as some inhibit while others enhance immune responses [61]. Using salmonid cell lines to silence or overexpress *defb3* and infecting these cells with IHNV may help to ascertain its role during IHNV infection. IHNV also upregulates *hepc* mRNA in the kidneys of Sockeye salmon (*Oncorhynchus nerka*); although not confirmed, this may be attributed to IL-6 signaling, such as in mammals [62,63]. IHNV also induces the expression of *hepc1* mRNA in the anterior kidney and skin of Sockeye salmon [64]. In the anterior kidney, *hepc1* gene expression is directly correlated with the viral load [64]. This suggests that *hepc1* may be involved in the control of IHNV replication. The research surrounding AMP gene expression during IHNV infection in other salmonids besides rainbow trout and Sockeye salmon remains unknown. Our lab has generated a continuous cell line from Arctic Char, known as ACBA, and has confirmed its susceptibly to VHSV and IHNV replication; however, the AMP gene expression profile of this cell line during infection remains unknown [65]. Given that various AMPs, including hepcidin, have been detected in Arctic Char at the transcript level, it is possible that ACBA may express AMPs [66]. Expanding our knowledge of which AMP genes are expressed across salmonid species and in what context will increase our understanding of the host response to viral disease.

Other viruses that infect salmonids include infectious pancreatic necrosis virus (IPNV); this virus is unique because it leads to substantial changes in iron metabolism within the head kidney via the upregulation of genes involved in iron metabolism, including hepcidin, and subsequently leading to oxidative stress [67]. Unlike IHNV, IPNV infection in Atlantic salmon does not increase *hepc* in the head kidney, but does upregulate *hepc* gene expression in liver and red blood cells. In addition to this, IPNV leads to an upregulation of *cath* mRNA in the head kidney and red blood cells of infected fish [67]. Interestingly, IPNV infection significantly increases the plasma levels of both Cath and Hepc [67], suggesting that AMPs may be involved in systemic responses to IPNV infection. Immunization against IPNV induces differential AMP gene expression among rainbow trout tissues [68]. For example, in head kidney samples from immunized animals, *leap2a*, *defb1*, *defb2*, and *defb4* transcripts increased, while in the pyloric ceca, only *leap2a* and *defb4* transcripts increased [68]. The pyloric ceca in rainbow trout has been identified to be a major site for B cell recruitment following vaccination, which may influence AMP gene expression in this organ [69]. However, the role of AMPs in vaccination against viruses remains understudied.

### 4.3. Parasites

Salmonids serve as a host for a multitude of parasites that may result in tissue damage and severe inflammatory responses [64,70,71,72,73]. It is not uncommon for co-infection to occur with other parasites or pathogens, thus leading to enhanced immune activation [63,72]. However, the impact of parasitic pathogens on AMP gene expression remains understudied. The myxozoan parasite, *Tetracapsuloides bryosalmonae* (*T. bryosalmonae*), also the causative agent of proliferative kidney disease (PKD), has been shown to modulate the expression of AMPs in brown trout [51]. During the response to *T. bryosalmonae* infection in the kidney, *defb3* was rapidly upregulated at the transcriptional level and this was sustained at 7 and 16 dpi [51]. Interestingly, fish that did not test positive for *T. bryosalmonae* also demonstrated higher than baseline levels of *defb3* gene expression, suggesting that it may confer protection against *T. bryosalmonae* [51]. Other AMP genes, such as *cath1* and *hepc*, were also significantly upregulated in the kidney, but only when the fish were co-infected with *T. bryosalmonae* and VHSV [51], thus providing evidence to support that, in some cases, co-infection with parasites leads to enhanced immune activation.

An important metazoan parasite of farmed and wild salmonids is *Lepeoptherius salmonis* (*L. salmonis*), more commonly known as sea lice [64]. *L. salmonis* infection results in an up-regulation of *hepc1* mRNA in the skin of Sockeye salmon [64]. On the other hand, sea lice such as *Caligus rogercresseyi* (*C. rogercresseyi*), elicit differential AMP gene expression profiles, based on relative transcript abundance, in the skin of Atlantic salmon and Coho salmon (*Oncorhynchus kisutch*) [74]. Although the *hepc* gene is expressed in both salmonid skin tissues, whether *C. rogercresseyi* infection has occurred or not, it appears to decrease, along with *cath1*, in Coho salmon skin at 14 dpi, compared with 7 dpi with *C. rogercresseyi* [74], whereas the expression of *cath2*, *nkl*, *defb3*, and *defb4* appears to increase 14 dpi in Coho salmon skin [74].

Infection of rainbow trout with the skin ciliate parasite, *Ichthyophthirius multifiliis* (*I. multifiliis*), resulted in a significant upregulation of *hepc* mRNA 8 days post-infection in the skin and gill, but was downregulated in the spleen [75]. Vaccination of rainbow trout against *I. multifiliis*, using DNA vaccines encoding plasmids for *I. multifiliis* antigens, resulted in increased expression of *hepc* mRNA in the liver of vaccinated fish; however, vaccination did not lead to significant protection against *I. multifiliis* [76]. The data suggest that the rainbow trout hepcidin gene is susceptible to modulation by *I. multifiliis* in various tissues and this might be true in other salmonids; however, this remains to be investigated. Therefore, parasites such as *T. bryosalmonae*, *L. salmonis*, *C. rogercresseyi*, and *I. multifiliis* may be important modulators of AMP gene expression in salmonids.

### 4.4. Oomycyte and Fungal Pathogens

Despite several known fungal or fungal-like (oomycyte) pathogens of fish, many remain to be studied in salmonids especially with respect to the activation of AMP gene expression [77]. To date, very few studies have investigated the interaction between fungal or oomycyte pathogens and salmonid AMP gene expression. The most well studied oomycete of salmonids is a pathogenic water mould known as *Saprolegnia parasitica* (*S. parasitica*) [78,79,80]. One such study used a panel of rainbow trout cell lines: RTG-2, RTGill, RTL, and RTS11, derived from the gonad, gill, liver, and monocyte/macrophage, respectively [78]. *S. parasitica* was applied to each cell line and AMP gene expression was assessed over 24 h [78]. It was found that, in the presence of *S. parasitica*, several AMP transcripts were upregulated, but some, such as *hepc* in RTG-2 and RTL, or *cath1* in RTG2, were not expressed at all [78]. In another study, rainbow trout head kidney leukocytes were treated with the alkali-insoluble fraction of the *S. parasitica* cell wall, which served as a PAMP [81]. The leukocytes significantly expressed *cath1* mRNA compared with controls after 6 h [81]. Thus, demonstrating that specific components of the pathogen can initiate AMP gene expression. Moreover, in Atlantic salmon infected with *S. parasitica*, several AMPs were detected in gill tissue and show differential expression over time [81]. In fish with absent lesions 3 days post-exposure, the expression of *defb1* was significantly higher than fish with lesions [81]. Therefore, *defb1* may be important in resistance against *S. parasitica* infection. Whether the in vitro AMP gene expression response of Atlantic salmon cell lines mimics the in vivo response to *S. parasitica* or *S. parasitica* PAMPs remains unknown. Collectively, the data suggest that *S. parasitica* may only initiate AMP gene expression in select cell lines or tissues. Therefore, more research is needed to better understand the role of pathogenic fungi in regulating AMP gene expression in salmonids.

### 4.5. Diet

Nutrigenomics, or how nutrients interact with genes, has been a growing area of research in aquaculture, but despite many advances in the field, the impacts on the health and immunity of animals remain to be explored [82]. The diet influences the expression and function of several AMPs and there is emerging evidence to suggest that this is true in teleosts as well [82]. Many salmonids undergo periods without active feeding; during overwintering or throughout the spawning run [83]. The post-starvation transcriptome analysis of Atlantic salmon liver tissue revealed decreases in the precursors of *hepc* and *leap2a* transcripts [84]. This may be owing to investing energy into survival rather than immune responses. A second transcriptome analysis performed on Atlantic salmon fed a mostly plant-based diet instead of fish meal revealed a significant downregulation of *hepc1* precursor gene expression in the liver [85]. In addition, feeding rainbow trout with a peptidoglycan rich diet resulted in downregulation of specific AMPs in the liver, gut, and gill tissue after 14 days, but increased AMP expression in the skin [85]. In another study, the withdrawal of peptidoglycan from the diet at day 14 resulted in decreased expression of *cath1*, *cath2,* and *leap2a*, but not *defb3* and *defb4* in the skin [86]. However, feeding peptidoglycan for 21–28 days continuously upregulates AMPs in rainbow trout skin, but eventually, the expression decreases [86]. Collectively, these findings suggest that continuous administration of peptidoglycan-enriched diets influence systemic AMP gene expression.

When rainbow trout were fed a protein-carbohydrate component of yeast cell wall (zymosan) enriched diet for 28 days, the expressions of *cath1* and *cath2* mRNA were found to be upregulated in the intestine of zymosan fed fish [28]. This was corroborated with in vitro evidence, whereby exposing the rainbow trout intestinal epithelial cell line, RTgutGC, to zymosan for 6 h resulted in an increase of *cath1* and *cath2* gene expression [28]. Zymosan exposure also induced the synthesis of Cath2 in the RTgutGC cell line at the protein level [28]. When the rainbow trout macrophage/monocyte cell line, RTS11, was incubated with vitamin C and heat-killed *E. coli*, the expression of *cath2* and *hepc* genes were significantly upregulated compared with controls [87]. However, this was not the case in rainbow trout head kidney leukocytes from fish exposed to the same conditions [87]. This suggests that not all leukocytes may respond to vitamin C and *E. coli* in the same manner as macrophages or monocytes. Furthermore, the composition of rainbow trout head kidney, in terms of leukocyte populations, has been shown to fluctuate over time [88].

Other examples of AMP genes being regulated through nutritional means include the following: a metabolite of leucine known as β-hydroxy-β-methylbutryic acid (HMB) that aids in preventing muscle atrophy and vitamin D [89,90,91]. For example, Atlantic salmon exposed to HMB in the water as well as in feed show upregulation of CATH2 in the gills [89]. In addition, HMB induces *cath2* and *hepc* gene expression in the embryonic-derived CHSE-214, Chinook salmon cell line [89]. Meanwhile, vitamin D treatment of the same cells only induces *cath2* [89]. This suggests that the *cath2* gene in Chinook salmon may have a vitamin D response element (VDRE) in the promoter region, but this may be lacking in the *hepc* gene within this species [91].

### 4.6. Development

Besides maternal antibodies in the egg, young salmonids may also depend on AMPs as a first line of defense as the immune system is immature [92,93]. Very few studies have examined AMPs in salmonid fry or larvae, but it is evident that some salmonids possess and activate the AMP defense system very early in development. The expression of *hepc* and *cath2* mRNA has been observed as early as 10 days post-hatch in rainbow trout exposed to *I. multifiliis* [94]. In rainbow trout larvae (17 days post-hatch) challenged with *Y. ruckeri*, *cath2* mRNA is significantly downregulated at 72 and 96 h post-challenge [95]. In rainbow trout fry (87 days post-hatch) challenged with *Y. ruckeri*, a rapid increase of *hepc* gene expression is observed within 24 h, but this is significantly downregulated after 25 days [95]. The expressions of other AMP genes, such as *cath2*, are upregulated later during the challenge, at 72 h, and also significantly decline after 25 days [95]. In addition, *defb1* mRNA was also found to significantly decrease at 25 days post-challenge [95]. The expressions of *hepc* and *cath1* were also observed at 34 and 42 days post-hatch in rainbow trout immersed in LPS for 8 h [96]. In the same fry, Hepc and Cath1 were detected via immunofluorescence in the skin, gut, and gill [96]. This suggests that *hepc*, *cath1*, and *cath2* may be involved in early immune responses in salmonids. Cell lines from salmonids are an incredibly powerful tool to investigate AMPs [2,97]. Despite the generation of several embryonic-derived cell lines from rainbow trout and other salmonids, one embryonic cell line to date has been used to investigate AMP expression in health and disease [89]. This cell line, known as CHSE-214, has been shown to express *hepc* and *cath2* [89]. Unfortunately, the embryonic stage of the animals from which CHSE-214 was derived is unknown, but the fact that the cell line retained its ability to express AMPs suggests that AMPs must play an important role in early salmonid immune responses or development. However, more research is needed to characterize the role of AMPs during early life stages in salmonids.

## 5. Immunomodulatory Role of Salmonid AMPs

### 5.1. Cathelicidins

Cathelcidins are a family of AMPs that, in addition to their antimicrobial activities, have been implicated in wound healing, angiogenesis, and other innate immune mechanisms [98]. Interleukin 8 (Il-8), a chemokine produced by macrophages and other leukocytes, has been proven to mediate stimulated migration of neutrophils, macrophages, peripheral blood leucocytes (PBLs), and head-kidney leucocytes (HKLs) in fish [99,100,101]. Cath1 and Cath2 stimulate PBLs to upregulate Il-8, but not Il-1β and interleukin 18 (Il-18), in Atlantic salmon at five and eight h post-infection (hpi) [24]. This was also the case for Cath1 and Cath2 in rainbow trout PBLs as early as 4 h post-incubation [98]. Further analysis of alternative splicing products from rainbow trout *cath1a* and *cath1d* genes showed that the ability to induce Il-8 in PBLs was dependent on structural flexibility in the central region, as Il-8 induction potential gradually decreased as structural flexibility was lost [98]. Atlantic salmon Cath applied to Atlantic salmon HKLs slightly induced interferon gamma (Ifn-γ), suggesting this peptide is involved in Ifn-γ-induced antigen presentation [102,103]. However, Il-8 stimulation could not be replicated, which might be owing to a deletion of a tandem repeat in the central region of the studied peptide compared with the previously studied variant, as this loss of structural flexibility was shown to have a significant impact on the immunomodulation ability of salmonid cathelicidins on Il-8 induction [24,103].

Salmonid cathelicidins were also found to stimulate phagocytosis. Synthetic mature cathelicidin peptides from brown trout, rainbow trout, and grayling enhanced phagocytic activity of phagocyte-enriched HKL cultures in a dose-dependent manner [27]. Moreover, incubation of HKLs with brown trout Cath1 slightly increased HKL reactive oxygen species (ROS) activity and had a synergic effect when incubated together with either β-glucan or phorbol 12-myristate 13-acetate [103]. Rainbow trout cathelicidins also stimulated phagocytic, ROS, and intracellular bactericidal activities of rainbow trout B cells, similar to LL-37, a mammalian AMP, in human macrophages [103,104]. A possible mediator of phagocytic activity in fish might be the purinergic receptor P2X 7 (*p2x7*). It was shown that *p2x7* plays a role in phagocytosis of fish macrophages [104]. Comparison of gene expression levels of this receptor, in phagocytic and non-phagocytic trout B cells, showed an upregulation in phagocytic B cells, suggesting this receptor might also be involved in B cell phagocytic activity [103]. In the rainbow trout gut epithelial cell line, RTgutGC, both variants of rainbow trout Cath2 induced *il1b* gene expression [45]. This stimulation was even stronger when β-glucan was added together with Cath2 while incubating for 6 h [24].

### 5.2. Defensins

Immunomodulation studies involving salmonid β-defensins are scarce. MX Dynamin-like GTPase 1 (*mx1*), an IFN-induced gene, was found to be upregulated eightfold in EPC cells that were transfected with a plasmid containing a recombinant rainbow trout *defb1* [35]. Whether this AMP plays a role in the antiviral immune response in rainbow trout remains to be tested. As aforementioned, myeloid rainbow trout B cells were also found to express *defb3* [98]. However, further research is needed to elucidate whether β-defensins can modulate innate immune responses in trout B cells. Aside from rainbow trout β-defensins, no other salmonid β-defensins were tested for their immunomodulatory activities as of the writing of this review. With the recent discovery of five β-defensin clusters in salmonids and our recent discovery of a novel variant of the rainbow trout β-defensin 1 gene in silico (unpublished), this family of AMPs holds great potential for further immunomodulation studies [45].

### 5.3. Hepcidins

Very few studies have been published concerning the immunomodulatory effects of hepcidin peptides in salmonids, despite that several hepcidins were identified in this group of fish [30,31,32,33,105]. As of the writing of this review, such immunomodulatory studies are limited to Caspian trout (*Salmo caspius*). Injection of 1 µg/g synthetic Caspian trout Hepc into healthy specimens induced the expression of pro-inflammatory cytokine genes in the spleen and kidney [105]. Twenty-four hours after injection, only interleukin 6 (*il6*) and *tnfa* gene expressions were significantly increased in the spleen, whereas in the kidney, all pro-inflammatory cytokines tested (*il1b*, *il6*, and *tnfa*) were significantly upregulated at the gene level [106]. IL-6 and others of the IL-6 subfamily of cytokines are known to be major players in haematopoiesis, and have pro- and anti-inflammatory properties [99,106,107]. Similarly, TNF-α and IL-1β, as pro-inflammatory cytokines, show overlapping functions as regulators of inflammation in fish [99]. These results suggest that presence of Caspian trout hepcidin at least induces pro-inflammatory immune responses in healthy specimens.

### 5.4. NK-lysins

Similar to defensins and hepcidins, the immunomodulatory functions of salmonid NK-lysins are relatively understudied. In a recent study, Acosta et al. (2019) analysed whether short peptides derived from Atlantic salmon NK-lysin 1 and 2, with proposed immunomodulatory activities, had an effect on the expression of a small selection of cytokines in Atlantic salmon HKLs [102]. Stimulation with 50 µM of both peptides induced expression of pro-inflammatory *il1b* and *il8* in HKLs [102]. These results suggest that NK-lysins might act as a signal to recruit immune cells similar to cathelicidins, as aforementioned. However, this hypothesis remains to be tested in salmonids.

## 6. Conclusions

Studies on the form and functionality of salmonid AMPs greatly contributed to the knowledge of their role in the innate immune system, but many questions remain unanswered. With the exception of piscidins, all major families of fish AMPs (cathelicidins, hepcidins, defensins, NK-lysins, and histone-derived AMPs) are present in salmonid fish. Important factors affecting salmonid AMP function are as follows: length, which is associated with structural flexibility; lower total hydrophobic ratio, which is correlated with lower hemolytic activity; and, although not essential, positive overall charge. Some families of salmonid AMPs (cathelicidins, defensins, and hepcidins) are better studied than others, such as histone-derived AMPs. Furthermore, much of the research to date has focused on economically important species such as rainbow trout and Atlantic salmon contributing to a lack of studies on other salmonid AMPs. Although the potent direct antimicrobial activities of salmonid AMPs have been well established in vitro, their direct antiviral, antiparasitic, and antifungal roles remain to be understood. A large body of evidence supports the expression of salmonid AMPs in response to various pathogens and stimuli including PAMPs, bacteria, viruses, parasites, fungi, diet manipulation, and even developmental stage. In general, the expression of AMPs either remains indifferent or is upregulated upon challenge with dead or live pathogens or pathogen components, but downregulated upon starvation of fish, which might be owing to an energy conservation strategy. However, differences in the transcriptional regulation of AMPs are found between species, between different immune-relevant organs, and even between different types of leukocytes. Salmonid AMPs appear to be rapidly induced at the transcript level in response to various pathogenic or dietary stimuli, but the various upstream molecular signaling pathways leading to this rapid response remain unknown. In addition, young salmonids show higher levels of AMP expression, compared with adults, suggesting that they rely more on the innate immune response at an early life stage. Apart from their aforementioned direct antimicrobial activities, salmonid AMPs have been shown to possess several immunostimulatory properties as well, such as stimulating the migration of leukocytes or increasing the phagocytic and ROS activity of immune cells. Salmonid AMPs are diverse molecules and play a functional role in the defense against pathogens. However, further research is needed to ascertain their role in salmonid health and disease. As the effects of stress and handling on salmonid AMP gene expression are also unknown, this may be a useful area of future research. Exposing salmonid cell lines to the stress hormone, cortisol, and assessing AMP expression may reveal interesting avenues of investigation for in vivo studies. The effects of climate change on AMP-dependent immunity in salmonids should also be investigated. Exposing fish to extremes may yield important information on how salmonids will cope in future environmental scenarios. This will be important for the future of aquaculture and wildlife biology as emerging pathogens and changing climates continue to wreak havoc on fish populations. Lastly, the discovery of novel salmonid AMPs may lead to new therapeutics in medicine and will help understand the complex evolutionary history of salmonid species.

## Supplementary Materials

The following are available online at https://www.mdpi.com/2079-7737/9/8/233/s1, Figure S1: Phylogenetic tree of published salmonid cathelicidins, Figure S2: Phylogenetic tree of published and predicted salmonid hepcidins, Table S1: Salmonid antimicrobial peptides from databases.
