# Supplementary material for: Antimicrobial Peptides of Salmonid Fish: From Form to Function
Source: Biology (Basel). 2020 Aug 18;9(8):233. doi: 10.3390/biology9080233 (PMC7464209; doi:10.3390/biology9080233)
Supplement: Supplementary file 1 [file biology-09-00233-s001.pdf]

biology

*Review*

# Antimicrobial Peptides of Salmonid Fish: From Form to Function

Sascha R. Brunner, Joseph F. A. Varga, and Brian Dixon

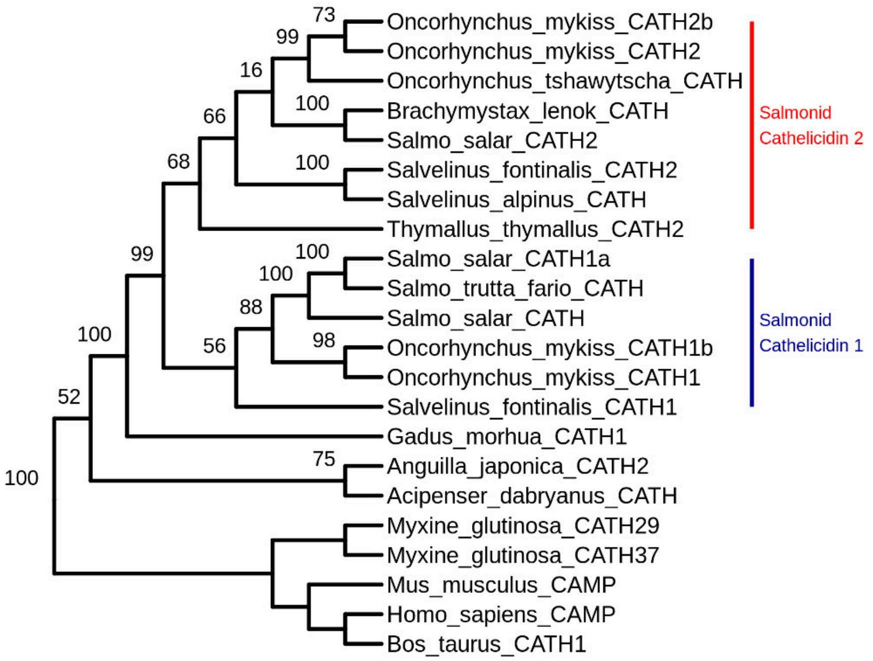


**Figure S1.** Phylogenetic tree of published salmonid cathelicidins. Cathelicidins form two well-supported clades within salmonids. The tree was constructed on exon 1-3 as exon 4 is highly variable. All analysis was done in R v3.6.1 (The R Core Team 2020). Amino acid sequences were retrieved from GenBank and aligned using ClustalW. A pairwise distance matrix was constructed with the pairwise deletion option selected. The tree was constructed with the improved neighbour-joining method BIONJ [1]. The tree is rooted with 3 mammalian and 2 agnatha sequences. Root nodes indicate bootstrap values (1000 replicates) in percent.

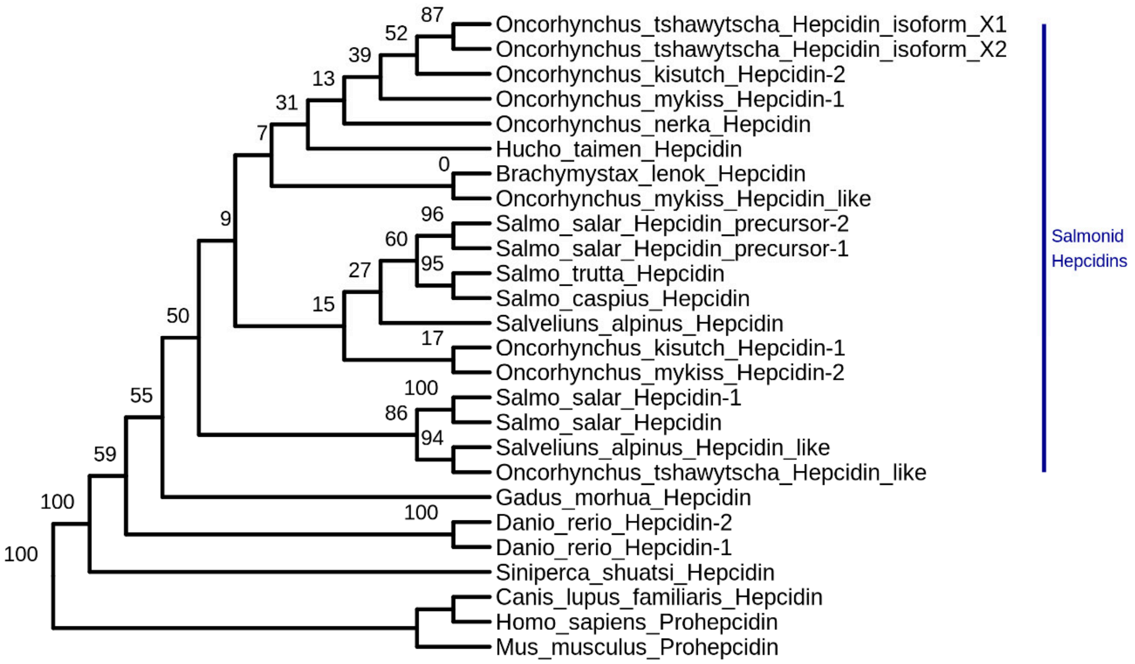


**Figure S2.** Phylogenetic tree of published and predicted salmonid hepcidins. Hepcidins form one clade within salmonids. For analysis details, see description of Figure S1. Amino acid sequences were retrieved from GenBank. The tree is rooted with 3 mammalian sequences. Root nodes indicate bootstrap values (1000 replicates) in percent.

**Table S1.** Salmonid Antimicrobial Peptides from Databases.

| ID | Name | Source | Sequence |
|---|---|---|---|
| DRAMP02344 | 40S ribosomal protein S30 | *O. mykiss* | KVHGSLARAGK |
| AP03058 | CK11 | *O. mykiss* | AIPKCCVGTSRNIPLSILMRVERYDVQHNHGACEIDAVVLHANGRKYCADPRVKKVLGVAMQIRKAQLMREKLNSIMRR |
| AP01326 | Oncorhyncin I | *O. mykiss* | SKGKKANKDVELARG |
| AP0041 | Oncorhyncin II | *O. mykiss* | KAVAAKKSPKKAKKPATPKKAAKSPKKVKKPAAAAKKAAKSPKKATKAAKPKAAKPKAAKAKKAAPKKK |
| AP00410 | Oncorhyncin III | *O. mykiss* | PKRKSATKGDEPARRSARLSARPVPKPAAKPKKAAAPKKAVKGKKAAENGDAKAEAKVQAAGDGAGNAK |
| DRAMP02334 | Hepcidin | *O. mykiss* | XSHLSLCRWCCNCCHNKGXGFCCKF |
| DRAMP02338 | Beta-defensin 1 | *O. mykiss* | MVTLVLLVFLLLNVVEDEAASFPFSCPTLSGVCRKLCLPTEMFFGPLGCGKGFLCCVSHF |
| DRAMP02339 | Histone H6-like protein | *O. mykiss* | PKRKSATKGDEPA |
| DRAMP18698 | Histone H2A | *O. mykiss* | AERVGAGAPVYL |
| DRAMP02340 | Salmocidin-1 | *O. mykiss* | XXSVPAFGHYLPAXP |
| DRAMP02341 | Salmocidin-2A | *O. mykiss* | SGFVLKGYTKTSQ |
| DRAMP02342 | Salmocidin-IIb | *O. mykiss* | AGFVLKGYTKTSQ |
| DRAMP02343 | Salmocidin-3 | *O. mykiss* | XXPQQLGHVKAAXSDY |
| DRAMP02345 | LEAP2A | *O. mykiss* | PEGQRALKRMARMTPLWRTMGTKPYGAYCLNNYECSTGICRGGHCMFSQPIKS |
| DRAMP02346 | LEAP2B | *O. mykiss* | GVCLVALILMHQVCASPIGSHDSRLSLQQGTKLLERRTRMTPLWRFMGTKPTGAYCRDHFECSTQICRRGHCALSGA |
| AP02535 | rtCATH-1a | *O. mykiss* | RRSKVRICSRGKNCVSRLGGGSIIGRPGGGSLIGRPGGGSVIGRPGGGSPPGGGSFNDEFIRDHSDGNRFA |
| AP02536 | rtCATH-1b | *O. mykiss* | RRSKVRICSRGKNCVSRPGGGSVIGRPGGGSPPGGGSFNDEFIRDHSDGNRFA |
| AP02537 | rtCATH-1c | *O. mykiss* | RRSKVRICSRGKNCVSRPGGGSFNDEFIRDHSDGNRFA |
| AP02538 | rtCATH-1d | *O. mykiss* | RRSKVRICSRGKNCVSFNDEFIRDHSDGNRFA |
| AP02539 | rtCATH-2a | *O. mykiss* | RRGKDSGGPKMGRKDSKGCWRGRPGSGSRPGFGSGIAGASGVNHVGTLPASNSTTHPLDNCKISPQ |
| AP02540 | rtCATH-2b | *O. mykiss* | RRGKDSGGPKMGRKDSKGCWRGRPGSGSRPGFGSGIAGASGVNHVGTLPA |
| DRAMP04588 | rtCATH-1 | *O. mykiss* | RICSRDKNCVSRPGVGSIIGRPGGGSLIGRPGGGSVIGRPGGGSPPGGGSFNDEFIRDHSDGNRFA |
| AP03053 | Bthepc | *S. trutta* | QSHLSLCRWCCNCCHNKGCGFCCKF |
| DRAMP02324 | SAMP H1 | *S. salar* | AEVAPAPAAAAPAKAPKKKAAAKPKKAGPS |
| DRAMP02313 | Hepcidin-1 | *S. salar* | QIHLSLCGLCCNCCHNIGCGFCCKF |
| AP02175 | CATH_BRALE | *B. lenok* | RRSKARGGSRGSKMGRKDSKGGSRGRPGSGSRPGGGSSIAGASRGDRGGTRNA |

Information in this table was obtained from The Antimicrobial Peptide Database (APD): http://aps.unmc.edu/AP/main.php and the Data Repository of Antimicrobial Peptides (DRAMP): http://dramp.cpu-bioinfor.org/.

## References

1. Gascuel, O. BIONJ: An improved version of the NJ algorithm based on a simple model of sequence data. *Mol. Biol. Evol.* **1997**, *14*, 685–695, doi:10.1093/oxfordjournals.molbev.a025808.

© 2020 by the authors. Licensee MDPI, Basel, Switzerland. This article is an open access article distributed under the terms and conditions of the Creative Commons Attribution (CC BY) license (http://creativecommons.org/licenses/by/4.0/).
